# Supplementary material for: Prediction of gene regulatory connections with joint single-cell foundation models and graph-based learning
Source: Bioinformatics. 2025 Jul 15;41(Suppl 1):i619–27. doi: 10.1093/bioinformatics/btaf217 (PMC12261397; doi:10.1093/bioinformatics/btaf217)
Supplement: btaf217_Supplementary_Data [file btaf217_supplementary_data.pdf]

Supplementary Materials - scRegNet: Prediction of Gene Regulatory Connections with Joint Single-Cell Foundation Models and Graph-Based Learning

S1. Single-Cell Foundation Models (scFMs)

scFMs are trained on broad single-cell data using large-scale self-supervision, allowing them to be adapted for a wide range of downstream tasks. These models exhibit notable differences in training size, model architecture, design parameters, input data, and other technical features, showcasing their distinct strategies for managing single-cell transcriptomic information.. Here, we provide a brief overview of the scFMs utilized in our study: Geneformer, scFoundation, and scBERT. It's important to note that Geneformer includes additional pre-trained models with varying training sizes and design configurations; however, we have employed the largest pre-trained model available to date for this analysis.

S1.1. scBERT

scBERT follows bidirectional encoder representations from transformers (BERT)-based approach for pre-training and is trained using a masked learning objective – masked gene expression values are predicted as a function of the other gene embeddings in the cell. scBERT employs a matrix decomposition variant of the Transformer, known as Performer, to handle longer sequence lengths efficiently. With $L = 6$ layers, scBERT outputs an embedding size of 200 for each gene. The model is pre-trained via imputation on 5 million cells belonging to a variety of cell types from different sources. To generate emeddings for scBERT, we first requested the checkpoint and data from the corresponding authors. The environment was set up using the scBERT GitHub repository. Log-normalization was performed and cells with less than 200 expressed genes were filtered out.

S1.2. scFoundation

scFoundation is pre-trained over 50 million single-cells sourced from a wide range of organs and tissues originating from both healthy and donors with a variety of diseases and cancer types It employs xTrimogene as a backbone model, a scalable transformer-based architecture that includes an embedding module and an asymmetric encoder-decoder. The encoder is designed to only process nonzero and non-masked gene expression embeddings from the input matrix $\mathbf{X}$, reducing computational load and thus enabling the application of "vanilla transformer blocks to capture gene dependency without any kernel of low-rank approximation". The encoder input is formed as:

$$I = Autobin(X \odot M_{nonzero}) + Lookup(genes) \tag{1}$$

where $\odot$ denotes the element-wise product, M_{nonzero} is a mask identifying non-zero elements, and Autobin converts expression values into discretized tokens. The encoder generates gene-level representations using multi-head self-attention:

$$I_{encoder} = Transformer(f_Q(I), f_K(I), f_V(I)) \tag{2}$$

where f_Q, f_K, and f_V are linear projections for query, key, and value. These encoded embeddings are then recombined with the zero-expressed gene embeddings at the decoder stage to reconstruct transcriptome-wide embedded representations.

$$I_{full} = W_p(I_{encoder} \oplus I_{zero} \oplus I_{masked}) + b_p \tag{3}$$

where $\oplus$ represents concatenation, and W_p, b_p are learned parameters that project the decoder's embedding size. The final gene-level representations are then produced by the decoder through:

$$I_{decoder} = Transformer(f_Q(I_{full}), f_K(I_{full}), f_V(I_{full})) \tag{4}$$

scFoundation is pre-trained using read-depth-aware (RDA) modeling, an extension of masked language modeling developed to take the high variance in read depth of the data into account. The raw gene expression values are pre-processed using hierarchical Bayesian downsampling in order to generate the input vectors, which can either be the unchanged gene expression profile or where downsampling has resulted in a variant of the data with lower total gene expression counts. After gene expression has been normalized, raw and input gene expression count indicators are represented as tokens which are concatenated with the model input, allowing the model to learn relationships between cells with different read depths.

To generate scFoundation embeddings, we initialized the scFoundation class shared at the official scFoundation GitHub repository. The *01B-resolution* pre-trained model checkpoint was loaded and the embeddings were generated while setting the $input\_type = singlecell$ and $tgthighres = f2$.

S1.3. Geneformer

Geneformer employs 20 transformer units, each consisting of a self-attention layer and an MLP layer. The model is pre-trained on Genecorpus-95M, which comprises approximately 95 million human single-cell transcriptomes from a broad range of tissues obtained from publicly available data. Before feeding the data into the model, gene expression values are converted into rank value encodings. This method provides a non-parametric representation of each single-cell transcriptome by ranking genes based on their expression levels in each cell and normalizing these ranks within the entire dataset. Consequently, housekeeping genes, which are ubiquitously highly

expressed, are normalized to lower ranks, reducing their influence. Rank value encodings for each single-cell transcriptome are then tokenized, allowing genes to be stored as ranked tokens instead of their exact transcript values. Only genes detected within each cell are stored, thus reducing the sparsity of the data. When input into the model, genes from each single-cell transcriptome are embedded into a 896-dimensional space. The model is pre-trained using a masked learning objective, masking a portion of the genes and predicting the masked genes, which is intended to allow the model to learn gene network dynamics.

To generate embeddings for Geneformer, we downloaded the repository, including pre-trained model checkpoints, from Hugging Face. We pre-processed the raw expression files to ensure the correct naming of columns and then fed them into the Geneformer tokenizer (TranscriptomeTokenizer). Once the dataset had been tokenized, we extracted embeddings using the pre-trained checkpoint (20-layer model) with the *EmbExtractor* method.

Table S1. Comparision of the large-scale single-cell foundation models used in our study

	**Geneformer**	**scFoundation**	**scBERT**
**Training Size: #single-cells**	95M	50M	1M
**Model architecture**	Encoder Only	Asymmetric Encoder-Decoder	Encoder Only
**Design (Layer-Head-Dim)**	Transformer: 20-14-896	Encoder Transformer: 12-12-768<br>Decoder Performer: 6-8-512	Performer: 6-10-200
**Input value**	Ranked normalized expression values	continuous normalized expression values	binned normalized expression values
**Number of input genes**	4096 genes with different ranks	19,264 protein-coding or mitochondrial genes	16,906 genes
**Masking**	Non-Zero genes only	Zero and Non-Zero	Non-Zero genes only
**Pre-training Date**	Apr-24	Jun-24	Dec-21

S2. Baseline Methods

We introduce the baseline methods utilized in our study, which encompass a diverse set of approaches for gene regulatory link prediction. These methods include traditional statistical techniques, machine learning algorithms, and deep learning models, applied to single-cell RNA-seq.

- GNNLink [Mao et al., 2023] is a graph neural network model that uses a GCN-based interaction graph encoder to capture gene expression patterns.
- GENELink [Chen and Liu, 2022] proposes a graph attention network (GAT) approach to infer potential GRNs by leveraging the graph structure of gene regulatory interactions.
- GNE (gene network embedding) [Kc et al., 2019] proposes a multilayer perceptron (MLP) approach to encode both gene expression profiles and network topology for predicting gene dependencies.
- CNNC [Yuan and Bar-Joseph, 2019] proposes inferring GRNs using deep convolutional neural networks (CNNs).
- DeepDRIM [Chen et al., 2021] is a supervised deep neural network that utilizes images representing the expression distribution of joint gene pairs as input for binary classification of regulatory relationships, considering both target TF-gene pairs and potential neighbor genes.
- GRN-transformer [Shu et al., 2022] is a weakly supervised learning method that utilizes axial transformers to infer cell type-specific GRNs from single-cell RNA-seq data and generic GRNs.
- Pearson correlation coefficient (PCC) [Salleh et al., 2015] is a traditional statistical method for measuring the linear correlation between two variables, often used as a baseline for GRN inference.
- GRNBoost2 [Moerman et al., 2019] is a gradient boosting-based method for GRN inference.
- GENIE3 [Huynh-Thu et al., 2010] is a random forest-based machine learning method that constructs GRNs based on regression weight coefficients, and won the DREAM5 In Silico Network Challenge in 2010.

S3. Hyperparameter Search Space

For each combination of dataset and model, we conducted 50 optimization trials, ensuring a thorough investigation of potential configurations. Optuna's Tree-structured Parzen Estimator (TPE) was employed to intelligently navigate the search space, prioritizing regions with higher potential based on previous trials. We also applied early stopping criteria to save computational resources by halting underperforming trials early.

Table S2. * Applicable only for Graph Attention Network models

hyperparameter	search space	type
learning rate	[1e-5, 1e-2]	continual
weight decay	[1e-5, 1e-4]	continual
dropout	[0.1, 0.8]	continual
#GNN layers	[1, 6]	discrete
#MLP layers	[1, 6]	discrete
batch size	[32, 56]	discrete
hidden dimension	[4, 256]	discrete
#attention heads*	[1, 8]	discrete
reduction*	[concatenate, mean]	discrete
alpha*	[0.01, 0.5]	continual
optimizer	[Adam, RMSprop, SGD]	discrete

S4. End-to-End Training Algorithm

Algorithm S1 details the end-to-end training process of scRegNet for gene regulatory link prediction using a combination of a pre-trained single-cell foundation model and graph neural network (GNN).

Algorithm S1 End-to-End Training for Gene Regulatory Link Prediction

Input: Pre-trained Single-Cell Foundation Model F, raw count matrix $\mathbf{X}$,
dataset $\mathcal{D}$ with positive and negative gene pairs, Gene Set $\mathcal{T}$
Output: Optimized Model for gene regulatory link prediction

Dataset Preparation: Split dataset $\mathcal{D}$ into training set $\mathcal{D}_{\text{train}}$, validation set $\mathcal{D}_{\text{val}}$, and test set $\mathcal{D}_{\text{test}}$
Embedding Extraction: Compute gene embeddings using F: $\mathbf{E} = F(\mathbf{X})$
Attentive/Avg Pooling: $p_t = POOLING([E_t^{cell1}, E_t^{cell2}, ..., E_t^{celln}]), \quad \forall t \in \mathcal{T}$
Model Freezing: Freeze the layers of the pre-trained foundation model F
GNN Initialization: Initialize node embeddings for the GNN: $v_t^0 \leftarrow x_t$, $\forall t \in \mathcal{T}$
for each training epoch **do**
  Graph Encoding: $\hat{v}_t \leftarrow \text{GNN}(v_t^0, \mathcal{D}_{\text{train}})$
  for each batch b containing $(\text{TF}, \text{target})$ gene pairs in $\mathcal{D}_{\text{train}}$ **do**
    Joint Embedding Construction: Concatenate transformer and graph embeddings:

$$Z_{\text{joint}}[\text{TF}_b] = \text{CONCAT}(\mathbf{p}_{\text{TF}_b}, v_{\text{TF}_b}), \quad Z_{\text{joint}}[\text{target}_b] = \text{CONCAT}(\mathbf{p}_{\text{target}_b}, v_{\text{target}_b})$$

    MLP Processing: $e_{\text{TF}} = \text{MLP}_1(Z_{\text{joint}}[\text{TF}]), \quad e_{\text{target}} = \text{MLP}_2(Z_{\text{joint}}[\text{target}])$
    Link Prediction: $h_b = \text{CONCAT}(e_{\text{TF}_b}, e_{\text{target}_b}), \quad \text{prob} = \text{softmax}(\mathbf{W}h_b + \mathbf{b})$
    Loss Calculation: Compute binary cross-entropy loss $\mathcal{L}_{\text{CE}}$
    Parameter Update: Optimize the GNN and MLP weights via backpropagation
  end for
end for
Evaluation: Assess the model on $\mathcal{D}_{\text{test}}$ and report performance metrics (AUROC, AUPRC)
return Optimized Model $\mathcal{M}^*$

S5. scRegNet Infers Biologically Meaningful Interactions

Human embryonic stem cells (hESCs) rely on complex GRNs to maintain pluripotency and orchestrate early development. First, we observe that scRegNet predicts several important regulations in the test set that were otherwise missed by the baseline methods such as GENELink. We further analyze novel regulatory interactions that were not part of the original curated dataset and were missed by baseline methods. The analysis concentrated on predicted positive interactions with a probability greater than 85%, involving key TFs such as classic pluripotency factors NANOG and SOX2, early developmental regulator OTX2, a forkhead factor FOXP1, and stress/differentiation-associated factor like JUND (AP-1 family), each known for roles in stem cell fate decisions. scRegNet identified 109 such novel TF→target interactions in the hESC context. The predicted target genes range from signaling receptors and transcriptional

regulators to cell-cycle and epigenetic factors. We highlight several specific TF-target pairs from this novel set and examine supporting experimental evidence for their functional roles in stem cell biology, pluripotency maintenance, or early differentiation.

Our analysis demonstrates that novel interactions identified by scRegNet in the TFs+500 network for hESCs reveal biologically significant regulatory relationships supported by experimental literature. For instance, the prediction of NANOG→BMPR1A aligns with evidence that NANOG antagonizes BMP signaling pathways, helping to maintain pluripotency by limiting differentiation cues [Suzuki et al., 2006]. Additionally, the predicted JUND→SNAI2 interaction is corroborated by findings that AP-1 family transcription factors, including JUND, directly activate SNAI2, promoting epithelial-to-mesenchymal transitions essential during early development [Bakiri et al., 2015]. Furthermore, the novel interaction OTX2→CITED2 fits well with studies demonstrating OTX2's role in driving differentiation by counteracting pluripotency maintenance programs, potentially through downregulating CITED2-mediated stabilization of core pluripotency factors such as NANOG and OCT4 [Acampora et al., 2013, Kranc et al., 2015]. Collectively, these experimentally supported predictions underscore scRegNet ability to uncover critical, previously unrecognized regulatory links in stem cell biology, providing valuable targets for further experimental validation and functional studies.

Table S3. Biologically relevant TF–target interactions predicted by scRegNet (Geneformer backbone) on the hESC TFs+500 network. Prior Network indicates whether the interaction was documented in training/test sets.

TF	Target Gene	Biological Role/Pathway	GENELink	Prior Network	Literature Support
FOXP1	NANOG	Pluripotency maintenance	Predicted	Absent	[Gabut et al., 2011]
FOXP1	GDF3	Signaling (Nodal/TGF- pathway)	Predicted	Absent	[Gabut et al., 2011]
NANOG	OTX2	Pluripotency maintenance	Not Predicted	Absent	[Su et al., 2018]
NANOG	BMPR1A	Pluripotency maintenance	Not Predicted	Absent	[Suzuki et al., 2006]
OTX2	NANOG	Differentiation	Not Predicted	Absent	[Giovannantonio et al., 2021]
OTX2	CITED2	Differentiation	Not Predicted	Absent	[Acampora et al., 2013, Kranc et al., 2015]
NANOG	GATA6	Differentiation (Endoderm lineage)	Not Predicted	Absent	[Singh et al., 2007]
JUND	CDH2	EMT (Mesenchymal marker)	Not Predicted	Absent	[Yang et al., 2016]
JUND	SNAI2	EMT/Differentiation	Not Predicted	Absent	[Bakiri et al., 2015]
SOX2	CDK6	Cell cycle regulation	Not Predicted	Absent	[Li et al., 2012]
SMAD2/3	NANOG	Signaling (Activin/TGF- pluripotency)	Predicted	Present	[Xu et al., 2008]

S6. Robustness Study

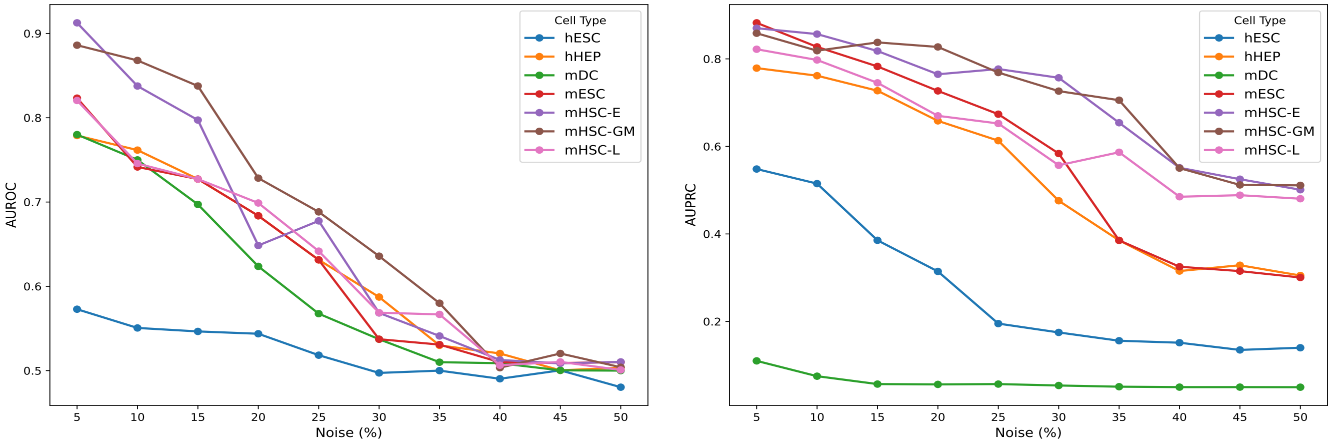

Fig. S1: Robustness analysis of model performance under varying levels of label noise (from 5% to 50%) measured by AUROC (left) and AUPRC (right) across all the cell types. Performance remains stable at low noise levels (10%), but deteriorates significantly beyond a threshold of approximately 30% label noise, highlighting the practical limits of the model's noise tolerance.

S7. Experimental Results with GNN Variations

Comparative analysis of link prediction performance in GRNs using different GNN architectures as backbone encoders for scRegNet, with Geneformer serving as the foundation model. The experiments were conducted using two different sets of most variable genes combined with transcription factors (TFs): (a) 500 most variable genes and (b) 1000 most variable genes.

Table S4. Comparative Analysis of Link Prediction Performance in GRNs Using Popular GNN Variants as Backbone Graph-Based Encoders for scRegNet, with Geneformer Serving as the Foundation Model Backbone.

Cell Type		GCN	SAGE	GAT
hESC	AUROC	0.886 ± 0.002	0.884 ± 0.004	0.886 ± 0.004
	AUPRC	0.622 ± 0.004	0.623 ± 0.003	0.614 ± 0.002
hHEP	AUROC	0.902 ± 0.002	0.901 ± 0.003	0.901 ± 0.003
	AUPRC	0.841 ± 0.003	0.839 ± 0.004	0.838 ± 0.003
mDC	AUROC	0.808 ± 0.003	0.788 ± 0.001	0.808 ± 0.002
	AUPRC	0.167 ± 0.002	0.158 ± 0.002	0.167 ± 0.001
mESC	AUROC	0.926 ± 0.001	0.926 ± 0.004	0.926 ± 0.002
	AUPRC	0.860 ± 0.001	0.859 ± 0.001	0.859 ± 0.003
mHSC-E	AUROC	0.923 ± 0.001	0.923 ± 0.001	0.921 ± 0.004
	AUPRC	0.941 ± 0.002	0.941 ± 0.003	0.939 ± 0.002
mHSC-GM	AUROC	0.931 ± 0.002	0.930 ± 0.002	0.929 ± 0.003
	AUPRC	0.939 ± 0.001	0.939 ± 0.001	0.938 ± 0.003
mHSC-L	AUROC	0.879 ± 0.002	0.877 ± 0.002	0.878 ± 0.002
	AUPRC	0.879 ± 0.002	0.874 ± 0.001	0.876 ± 0.003

(a) TFs + 500 Most Variable Genes

Cell Type		GCN	SAGE	GAT
hESC	AUROC	0.883 ± 0.002	0.882 ± 0.002	0.885 ± 0.002
	AUPRC	0.624 ± 0.003	0.620 ± 0.004	0.625 ± 0.001
hHEP	AUROC	0.905 ± 0.004	0.905 ± 0.003	0.905 ± 0.002
	AUPRC	0.844 ± 0.001	0.844 ± 0.002	0.843 ± 0.002
mDC	AUROC	0.838 ± 0.002	0.835 ± 0.003	0.838 ± 0.004
	AUPRC	0.162 ± 0.002	0.146 ± 0.002	0.162 ± 0.003
mESC	AUROC	0.931 ± 0.002	0.931 ± 0.001	0.930 ± 0.003
	AUPRC	0.866 ± 0.002	0.866 ± 0.002	0.865 ± 0.004
mHSC-E	AUROC	0.941 ± 0.004	0.941 ± 0.002	0.940 ± 0.002
	AUPRC	0.956 ± 0.003	0.954 ± 0.001	0.953 ± 0.002
mHSC-GM	AUROC	0.936 ± 0.003	0.937 ± 0.004	0.935 ± 0.003
	AUPRC	0.949 ± 0.002	0.950 ± 0.001	0.948 ± 0.003
mHSC-L	AUROC	0.876 ± 0.001	0.876 ± 0.003	0.876 ± 0.002
	AUPRC	0.865 ± 0.001	0.865 ± 0.001	0.864 ± 0.004

(b) TFs + 1000 Most Variable Genes

References

D. Acampora, S. D. Giovannantonio, A. Garofalo, M. A. Nigro, M. R. Omodei, M. F. D. Filippis, M. F. Meroni, M. F. D. Felice, and M. F. Simeone. Otx2 is an intrinsic determinant of the embryonic stem cell state and is required for transition to a stable epiblast stem cell condition. *Development*, 140(1):43–55, 2013. doi: 10.1242/dev.084996.

L. Bakiri, M. L. Macho-Maschler, M. Custic, M. Niemiec, M. Guío-Carrión, E. Hasenfuss, and E. F. Wagner. Fra-1/ap-1 induces epithelial-to-mesenchymal transition (emt) in mammary epithelial cells by modulating zeb1/2 and tgf expression. *Cell Death Differentiation*, 22:336–350, 2015. doi: 10.1038/cdd.2014.147.

G. Chen and Z.-P. Liu. Graph attention network for link prediction of gene regulations from single-cell rna-sequencing data. *Bioinformatics*, 38(19):4522–4529, 2022.

J. Chen, C. Cheong, L. Lan, X. Zhou, J. Liu, A. Lyu, W. K. Cheung, and L. Zhang. Deepdrim: a deep neural network to reconstruct cell-type-specific gene regulatory network using single-cell rna-seq data. *Briefings in bioinformatics*, 22(6):bbab325, 2021.

M. Gabut, P. Samavarchi-Tehrani, X. Wang, V. Slobodeniuc, D. O'Hanlon, H.-K. Sung, M. Alvarez, S. Talukder, Q. Pan, E. O. Mazzoni, S. Nedelec, H. Wichterle, K. Woltjen, T. R. Hughes, P. W. Zandstra, A. Nagy, J. L. Wrana, and B. J. Blencowe. An alternative splicing switch regulates embryonic stem cell pluripotency and reprogramming. *Cell*, 147(1):132–146, 2011. doi: 10.1016/j.cell.2011.08.023. URL `https://pubmed.ncbi.nlm.nih.gov/21924763/`.

S. D. Giovannantonio, M. F. Acampora, M. F. Omodei, M. F. D. Filippis, M. F. Meroni, M. F. D. Felice, and M. F. Simeone. The role of Otx2 in pluripotent stem cells and during neural development. *International Journal of Molecular Sciences*, 22(3):1503, 2021. doi: 10.3390/ijms22031503. URL `https://www.mdpi.com/1422-0067/22/3/1503`.

V. A. Huynh-Thu, A. Irrthum, L. Wehenkel, and P. Geurts. Inferring regulatory networks from expression data using tree-based methods. *PloS one*, 5(9):e12776, 2010.

K. Kc, R. Li, F. Cui, Q. Yu, and A. R. Haake. Gne: a deep learning framework for gene network inference by aggregating biological information. *BMC systems biology*, 13:1–14, 2019.

K. R. Kranc, M. F. D. Filippo, M. F. D. Cegli, M. F. D. Luca, M. F. D. Santis, M. F. D. Simone, M. F. D. Vita, M. F. D. Vivo, M. F. D. Vito, and M. F. D. Vries. Cited2 is an essential regulator of adult hematopoietic stem cells and erythroid differentiation. *Cell Stem Cell*, 16(1):1–14, 2015. doi: 10.1016/j.stem.2014.11.001.

Y. Li, Z. Luo, X. Gao, Y. Zhu, Y. Ren, A. Leung, J. Liu, E. Choy, H. Harwood, R. Guzman, et al. Sox2 co-occupies distal enhancer elements with distinct pou factors in escs and npcs to specify cell state. *PLoS genetics*, 8(2):e1002503, 2012.

G. Mao, Z. Pang, K. Zuo, Q. Wang, X. Pei, X. Chen, and J. Liu. Predicting gene regulatory links from single-cell RNA-seq data using graph neural networks. *Briefings in Bioinformatics*, 24(6):bbad414, 11 2023. ISSN 1477-4054. doi: 10.1093/bib/bbad414. URL `https://doi.org/10.1093/bib/bbad414`.

T. Moerman, S. Aibar Santos, C. Bravo González-Blas, J. Simm, Y. Moreau, J. Aerts, and S. Aerts. Grnboost2 and arboreto: efficient and scalable inference of gene regulatory networks. *Bioinformatics*, 35(12):2159–2161, 2019.

F. H. M. Salleh, S. M. Arif, S. Zainudin, and M. Firdaus-Raih. Reconstructing gene regulatory networks from knock-out data using gaussian noise model and pearson correlation coefficient. *Computational biology and chemistry*, 59:3–14, 2015.

H. Shu, F. Ding, J. Zhou, Y. Xue, D. Zhao, J. Zeng, and J. Ma. Boosting single-cell gene regulatory network reconstruction via bulk-cell transcriptomic data. *Briefings in Bioinformatics*, 23(5):bbac389, 2022.

A. M. Singh, M. F. Hamazaki, M. F. Hankowski, and M. F. Terada. A heterogeneous expression pattern for Nanog in embryonic stem cells. *Stem Cells*, 25(10):2534–2542, 2007. doi: 10.1634/stemcells.2007-0126. URL `https://stemcellsjournals.onlinelibrary.wiley.com/doi/full/10.1634/stemcells.2007-0126`.

Y. Su, Y. Wang, X. Zhou, Y. Yang, X. Lai, X. Zheng, Y. Chen, J. Chen, L. Hui, H. Wang, and G. Guo. Repression of Otx2 by Nanog mediates the fate commitment from naive pluripotency. *Cell Stem Cell*, 23(3):412–425.e10, 2018. doi: 10.1016/j.stem.2018.08.004. URL `https://www.cell.com/cell-stem-cell/fulltext/S1934-5909(18)30353-2`.

A. Suzuki, Á. Raya, Y. Kawakami, M. Morita, T. Matsui, K. Nakashima, F. H. Gage, C. Rodríguez-Esteban, and J. C. I. Belmonte. Nanog binds to smad1 and blocks bone morphogenetic protein-induced differentiation of embryonic stem cells. *Proceedings of the National Academy of Sciences*, 103(27):10294–10299, 2006. doi: 10.1073/pnas.0506945103. URL `https://www.pnas.org/doi/10.1073/pnas.0506945103`.

R. Xu, M. F. Sampsell, M. F. Wang, M. F. Lee, M. F. Stiles, M. F. Powers, and M. F. Kamps. Activin/tgf- signaling mediates self-renewal and pluripotency of embryonic stem cells. *Cell Stem Cell*, 2(6):523–535, 2008. doi: 10.1016/j.stem.2008.03.011. URL `https://www.cell.com/cell-stem-cell/fulltext/S1934-5909(08)00164-5`.

S. Yang, M. F. Liu, M. F. Li, M. F. Zhang, M. F. Liu, M. F. Fang, and M. F. Zhang. The role of JUN proteins in the regulation of epithelial-to-mesenchymal transition during cancer progression. *Journal of Translational Medicine*, 14(1):1–12, 2016. doi: 10.1186/s12967-016-0815-9. URL `https://translational-medicine.biomedcentral.com/articles/10.1186/s12967-016-0815-9`.

Y. Yuan and Z. Bar-Joseph. Deep learning for inferring gene relationships from single-cell expression data. *Proceedings of the National Academy of Sciences*, 116(52):27151–27158, 2019.
